# Bardet–Biedl syndrome: a case series

**DOI:** 10.1186/s13256-022-03396-6

**Published:** 2022-04-29

**Authors:** Omer Ali Mohamed Ahmed Elawad, Mumen Abdalazim Dafallah, Mohammed Mahgoub Mirghani Ahmed, Ahmed Abdalazim Dafallah Albashir, Sahar Mohammed Abbas Abdalla, Habiballa Hago Mohamed Yousif, Anwar Ali Elamin Daw Elbait, Moawia Elbalal Mohammed, Hassan Ismail Hassan Ali, Mohamed Mutasim Mohamed Ahmed, Najla Fouad Nassir Mohammed, Fadwa Hashim Mohamed Osman, Mussab Alnazeer Yousif Mohammed, Ejlal Ahmed Ebrahim Abu Shama

**Affiliations:** 1 Gezira Hospital for Renal Disease and Surgery, Wad Medani, Sudan; 2 Faculty of Medicine, University of Gezira, Wad Medani, Sudan; 3 Faculty of Medicine and Health Sciences, Omdurman Islamic University, Omdurman, Sudan; 4 Wad Medani Teaching Hospital, Wad Medani, Sudan; 5 Wad Medani Heart Disease and Surgery Center, Wad Medani, Sudan; 6 Saim Hospital of Ophthalmology, Wad Medani, Sudan

**Keywords:** Bardet–Biedl syndrome, Cilioapathy, Sudan

## Abstract

**Background:**

Bardet–Biedl syndrome is a rare multisystem autosomal recessive disorder that falls under the spectrum of ciliopathy disorders. It is characterized by rod–cone dystrophy, renal malformations, polydactyly, learning difficulties, central obesity, and hypogonadism. Many minor features that are related with Bardet–Biedl syndrome might aid in diagnosis and are crucial in clinical management. Bardet–Biedl syndrome is diagnosed on the basis of clinical signs and symptoms, which can be confirmed by genetic testing. Here we present four cases of Bardet–Biedl syndrome. To our knowledge, these are the first cases of Bardet–Biedl syndrome reported from Sudan.

**Case presentation:**

Here, we report four Sudanese patients who presented with a variety of clinical manifestations of Bardet–Biedl syndrome (two males, 50 and 16 years old; two females, 38 and 18 years old). The first two patients presented with features of chronic kidney disease. The third patient had recently been diagnosed with type 1 diabetes and diabetic ketoacidosis. The fourth patient showed signs of retinal dystrophy early on. Case 1: a 38-year-old female presented with vomiting and irritability; the patient was diagnosed with Bardet–Biedl syndrome as she fulfilled six items of the primary features (obesity, retinitis pigmentosa, post-axial polydactyly, renal abnormalities, learning disabilities, and genitourinary malformations), as well as one secondary feature (cardiovascular involvement, that is, left ventricular hypertrophy). Case 2: a 50-year-old male presented with fatigability; the patient was diagnosed with Bardet–Biedl syndrome as he fulfilled four items of the primary features (obesity, retinitis pigmentosa, post-axial polydactyly, and renal abnormalities) in addition to two secondary features (diabetes mellitus and cardiovascular involvement, that is, left ventricular hypertrophy). Case 3: an 18-year-old female presented with polyuria, polydipsia, weight loss, and epigastric pain for 2 days; the patient was diagnosed with Bardet–Biedl syndrome because he had four major features (retinal dystrophy, post-axial polydactyly, obesity, and learning disabilities) in addition to three secondary features (developmental delay, diabetes mellitus, and strabismus). Case 4: a 16-year-old male presented with a blurring of vision; the patient was diagnosed with Bardet–Biedl syndrome as he exhibited four major features (retinal dystrophy, post-axial polydactyly, obesity, and learning disabilities) plus two secondary features (developmental delay and cataract).

**Conclusion:**

The scarcity of Bardet–Biedl syndrome necessitates a high index of suspicion to diagnose this syndrome. Increased awareness among physicians is required for the early diagnosis and treatment of Bardet–Biedl syndrome and to avoid complications and mortality.

## Background

Bardet–Biedl syndrome (BBS) is a rare, genetic multisystem disorder due to dysfunction of the primary cilia [1, 2]. This ciliopathy is characterized by rod–cone dystrophy, renal malformations, polydactyly, learning difficulties, central obesity, and hypogonadism [3]. It was first described by Bardet [4] in 1920 and then by Biedl [5] in 1922. Its prevalence in Europe and North America is 1:100,000 [6]. The incidence of this syndrome is higher in the Faroe Islands and Kuwait, with 1:3700 and 1:17,000 live births, respectively [7]. In Sudan, neither the incidence nor the prevalence of BBS are known. Twenty-one disease-causing genes have been detected so far (BBS1–BBS21). Owing to genetic heterogeneity and the excessive cost of genetic studies, they are usually restricted to difficult cases and research purposes only.

According to the diagnostic criteria published by Beales *et al*., the diagnosis of BBS is based on the presence of at least four primary features or three primary features and at least two secondary features [4] (Table 1). Genetic analysis for confirmation of BBS is not available in many resource-limited hospitals in developing countries. The rarity of the syndrome, as well as its slowly progressive course, poses a significant challenge for early diagnosis. Late detection can result in a higher rate of morbidity and mortality. Early detection of BBS is critical for halting the progression of renal impairment since it is the leading cause of morbidity and mortality in patients with BBS. The management of BBS is supportive through a multidisciplinary team approach. Family genetic counseling is critical.

**Table 1:** Diagnostic criteria for Bardet–Biedl syndrome (BBS)

Primary features	Case 1	Case 2	Case 3	Case 4	Secondary features	Case 1	Case 2	Case 3	Case 4
Truncal obesity	+	+	+	+	Strabismus/cataract/astigmatism	−	−	+	+
Retinitis pigmentosa/retinal dystrophy	+	+	+	+	Speech disorders/delay	−	−	−	−
Polydactyly	+	+	+	+	Developmental delay	−	−	+	+
Learning disabilities	+	−	+	+	Brachydactyly/syndactyly	−	−	−	−
Renal malformations	+	+	−	−	Behavioral disorders	−	−	−	−
Genital abnormalities (female) Hypogonadism (male)	+	−	−	−	Diabetes mellitus	−	+	+	−
					Polyuria/polydipsia (diabetes insipidus)	−	−	−	−
					Left ventricular hypertrophy (LVH) Congenital cardiac abnormalities	+	+	−	−
					Hepatic fibrosis	−	−	−	−
					Anosmia	−	−	−	−
					Craniofacial dysmorphic	−	−	−	−
					Dental crowding/high-arched palate/hypodontia/small roots	−	−	−	−
					Hirschsprung disease	−	−	−	−
					Ataxia/poor coordination	−	−	−	−

*LVH*: Left Ventriular Hypertrophy

## Case presentation

### Case 1

**Fig. 1:**
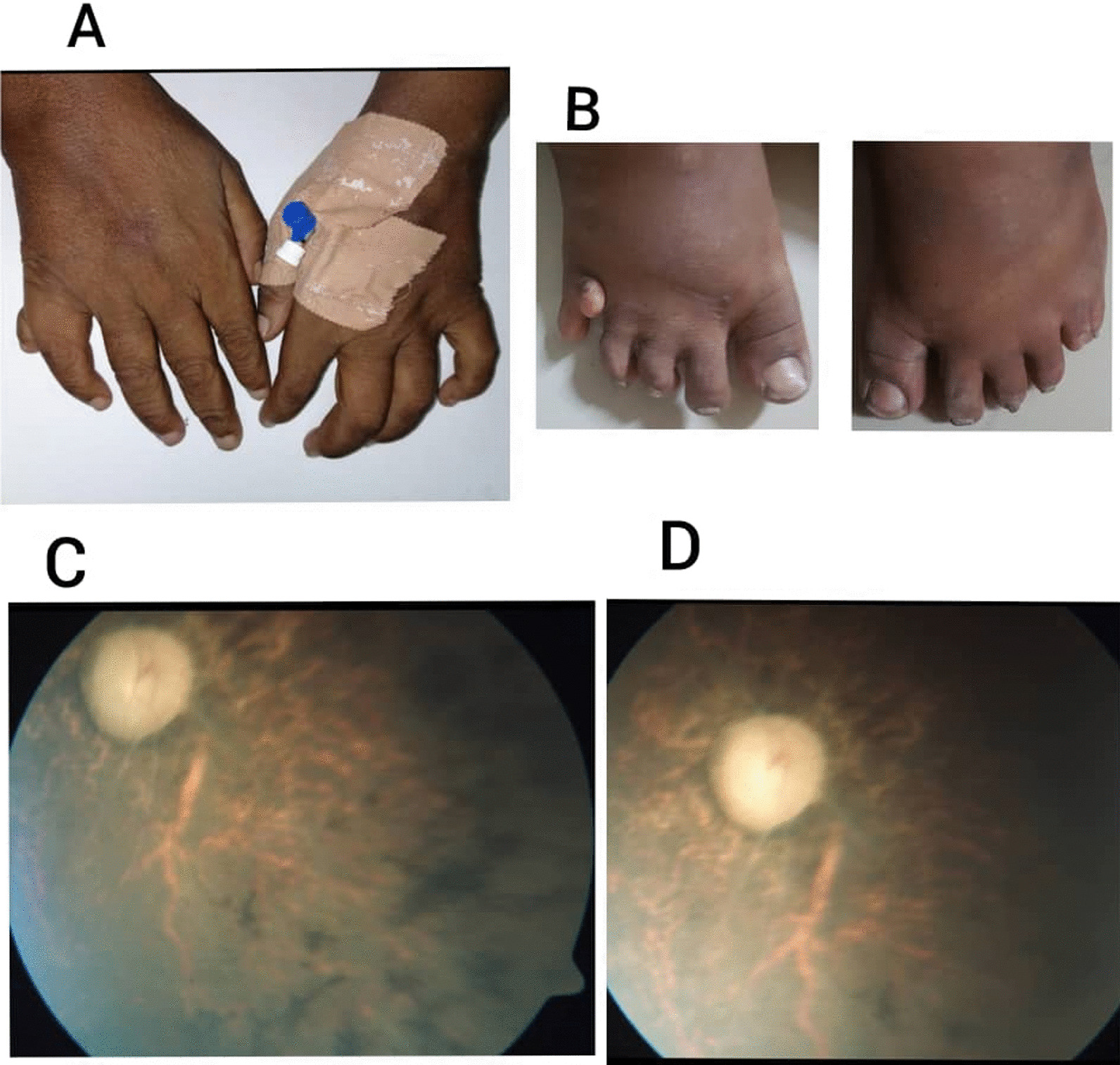
**A** Right upper limb post-axial polydactyly. **B** Bilateral lower limbs post-axial polydactyly. **C**, **D** Funduscopic pictures of both eyes showing retinitis pigmentosa and optic atrophy

A 38-year-old Sudanese single woman, the fifth issue of a consanguineous marriage, came to a university hospital in Sudan, complaining of irritability and vomiting. She had delayed developmental milestones and low school performance compared with her peers. Cognitive impairment was noticeable in terms of impaired perceptual reasoning, attention capacity, and functional independence. The patient started to experience night blindness at the age of 6 years, and by the age of 16, she had entirely lost her vision. There was no previous history of diabetes mellitus or hypertension. Her gynecological history revealed that she had menarche at the age of 14 and has had irregular cycles since then. On physical examination, her pulse was 80 beats per minute, her blood pressure was 130/80 mmHg, and she was pale. Her height and weight were 170 cm and 100 kg, respectively, with a body mass index (BMI) of 34.6 kg/m^2^, which indicates obesity. Fundus examination revealed retinitis pigmentosa and optic atrophy (Fig. 1C, D). The thyroid gland was not enlarged. During a musculoskeletal system assessment, it was discovered that there was post-axial polydactyly in the right upper limb (Fig. 1A) and bilateral lower limbs (Fig. 1B). The liver was enlarged to 4 cm below the right costal margin. Precordial and chest examinations were noncontributory.

Laboratory investigations showed serum sodium 110 mmol/L, blood urea 114 mg/dL, serum creatinine 6.2 mg/dL, and hemoglobin 6.4 g/dL (normochromic normocytic anemia). The glomerular filtration rate (GFR) value, based on four variables (age, race, gender, and plasma creatinine), was 8 mL/min/1.73 m^2^.

Abdominal ultrasonography showed a small right kidney (83 × 32 mm), while the left kidney was not detected. It also revealed an enlarged liver (16 cm) with a hyperechoic focal lesion (suggestive of hepatic hemangioma) and an infantile uterus. Electrocardiography showed features of left ventricular hypertrophy, which was confirmed by transthoracic echocardiography.

The diagnosis of BBS was made because the patient had six primary features plus one secondary feature.

(For more information on laboratory and imaging studies, see Table 2).

**Table 2:** The table above describes the findings of the laboratory investigations and imaging studies done for the patients

Laboratory investigations	Case 1	Case 2	Case 3	Case 4
CBC	Hb 6.4 g/dL (normochromic normocytic)	Hb 5.9 g/dL (normochromic normocytic)	Normal	Normal
RFT	BUN 114 mg/dL Serum creatinine 6.2 mg/dL	BUN 140 mg/dL Serum creatinine 4.3 mg/dL	Normal	Normal
RBS	180 mg/100 mL	210 mg/100 mL	388 mg/100 mL	110 mg/100 mL
HbA1C	5.9%	13%	14.5%	5.1%
TFT	Subclinical hypothyroidism	Normal	Normal	Normal
Serum electrolytes	Serum Na 110 (135–145) mmol/L Serum Ca 7.3 (8–11) mg/dL Serum PO_4_ 5.5 (3.4–4.5) mg/dL	Serum Ca 7.1 (8–11) mg/dL Serum PO_4_ 6.1 (3.4–4.5) mg/dL	Serum K 2.4 mmol/L	Normal
Serum uric acid	13.3 (2.6–5.7) mg/dL	8 (2.6–5.7) mg/dL	Normal	Normal
PTH	454 (15–75) pg/mL	490 (15–75) pg/mL	Normal	Normal
Urine general	Normal	Normal	++++ Acetone ++++ Sugar	Normal
Lipid profile	High serum cholesterol	Normal	Normal	Normal
Hormonal profile	Hypogonadotropic hypogonadism	Low testosterone	Normal	Normal
Imaging				
Abdominal ultrasonography	Small size right kidney Left kidney not detected Enlarged liver (hemangioma) Gall bladder stone Infantile uterus	Bilateral small kidney size	Normal	Enlarged liver
Echocardiography	Left ventricular hypertrophy (LVH)	Left ventricular hypertrophy (LVH)	Normal	Mild pulmonary HTN

*CBC*: Complete Blood Count; *Hb*: Haemoglobin; *RFT*: Renal Function Test; *BUN*: Blood Urea Nitrogen; *RBS*: Random Blood Sugar; *TFT*: Thyroid Function Test; *Serum Na*: Serum Sodium; *Serum Ca*: Serum Calcium; *Serum PO*_*4*_: Serum Phosphate; *Serum K*: Serum Potassium; *PTH*: Parathyroid Hormone; *LVH*: Left Ventriular Hypertrophy; *HTN*: Hypertention

The patient was transfused with three units of packed red cells and received thyroxine 25 μg once daily and allopurinol 100 mg OD. In addition to the fluid and salt restriction plans, she was given hypertonic saline (1 mL/kg/hour) for 48 hours and a proton pump inhibitor. Genetic counseling and the possibility of renal replacement therapy were discussed with her family.

The patient was discharged in good condition after day 5. Her parameters of red blood cells and serum sodium improved. Haemoglobin (HGB) was 9 g/dL, and serum sodium was 135 mmol/L. Erythropoietin injections, 1-alfacalcidol, folic acid, iron, calcium carbonate, thyroxine, and allopurinol tabs were prescribed before she was discharged. On her last follow-up, the patient was free of symptoms; her hemoglobin was 11 mg/dL, her blood urea was 70 mg/dL, and her serum creatinine was 3 mg/dL.

### Case 2

A 50-year-old Sudanese single man presented himself to a university hospital in Sudan, complaining of easy fatigue. The patient began to develop night blindness at the age of 7 years, and by the age of 15, he had entirely lost his vision. He had been diagnosed with diabetes mellitus for the preceding 6 years, and he had been managing it with insulin therapy and was experiencing frequent attacks of hypoglycemia. There was no history of hypertension or other chronic diseases. There is a history of polydactyly and visual impairment in the family.

**Fig. 2:**
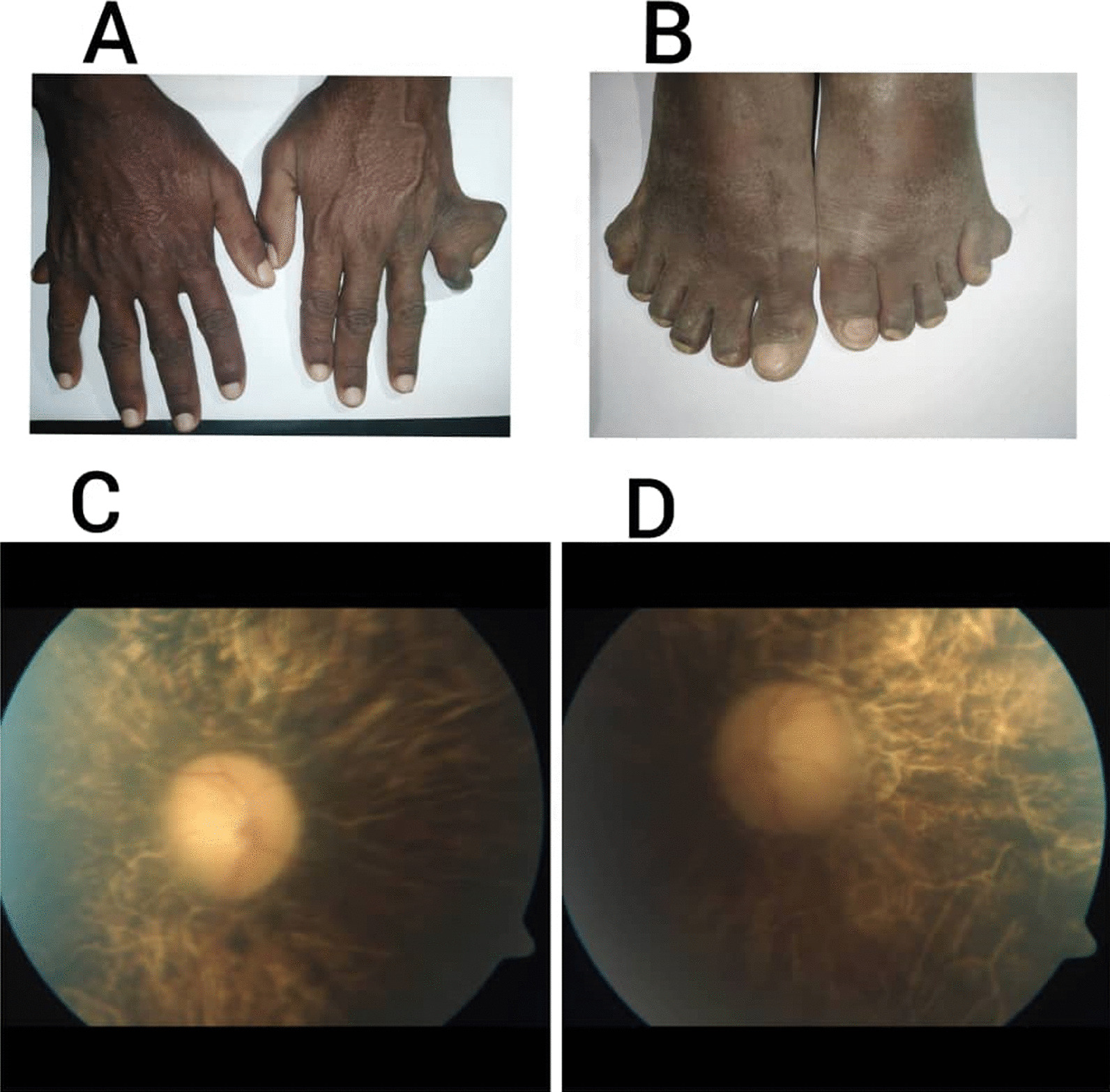
**A** Bilateral upper limbs post-axial polydactyly. **B** Bilateral lower limbs post-axial polydactyly. **C**, **D** Funduscopic pictures of both eyes showing retinitis pigmentosa

On physical examination, the patient was pale. He was 175 cm tall and 96 kg heavy, with a BMI of 31.3 kg/m^2^, indicating obesity. His vital signs were normal. Fundus examination showed features of retinitis pigmentosa (Fig. 2C, D). Examination of the extremities showed post-axial polydactyly in both the upper (Fig. 2A) and lower limbs (Fig. 2B).

Laboratory investigations showed normochromic normocytic anemia and a high renal profile. The glomerular filtration rate (GFR) was 17 mL/min/1.73 m^2^ based on four variables (age, race, gender, and plasma creatinine). Abdominal ultrasonography revealed bilateral small kidney size, and echocardiography showed left ventricular hypertrophy. The diagnosis of BBS was made on the basis of the presence of four items of primary features (obesity, retinitis pigmentosa, post-axial polydactyly, and renal abnormalities) in addition to two secondary features (diabetes mellitus and cardiovascular involvement, that is, left ventricular hypertrophy).

For more information on laboratory and imaging studies, see Table 2.

The patient was transfused with four units of packed red cells, and his insulin was stopped. The patient was discharged in good health. His indices of red blood cells improved; HGB was 10 g/dL; he was discharged on erythropoietin injections, 1-alfacalcidol, folic acid, iron, and calcium carbonate tabs.

At his last follow-up, the patient was free of symptoms.

### Case 3

An 18-year-old Sudanese girl, the third issue of inbred marriage, presented to a university hospital with complaints of polyuria, polydipsia, and weight loss for the previous 3 months, as well as epigastric pain for 2 days.

Compared with her siblings, she was behind on developmental milestones, including walking and speaking. She had a learning disability and poor academic performance as she is still in grade 4 primary school. When she was 8 years, she started to develop night blindness, and by the time she was 16, she had completely lost her sight. There had been no previous history of hypertension, chronic kidney disease (CKD), or other chronic illnesses. Menarche was at the age of 14 years, and since then she has had a regular cycle. Her family history was notable for her brother’s obesity, learning difficulties, six digits on three limbs, and visual impairment. In addition, her mother has diabetes mellitus.

**Fig. 3:**
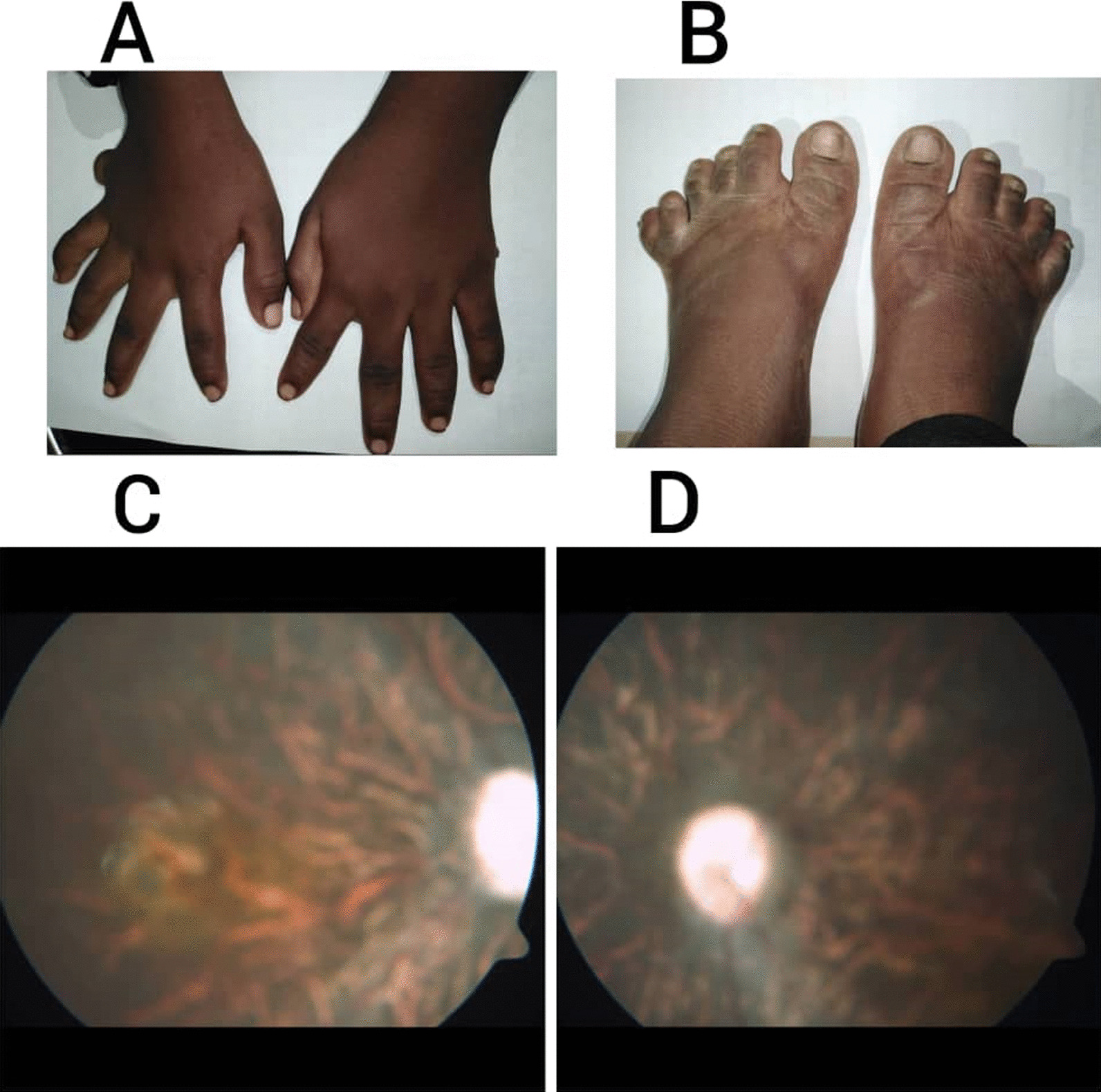
**A** Bilateral upper limbs post-axial polydactyly, **B** left lower limb post-axial polydactyly. **C**, **D** Funduscopic pictures of both eyes showing retinal dystrophy

Physical examination revealed a weight of 42 kg and height of 125 cm, with a BMI of 31.1 kg/m^2^, which indicates obesity. Her vital signs were normal. On the Wechsler adult intelligence scale, she received a score of 70, indicating mental retardation. Strabismus in her right eye was noticed. Fundus examination showed bilateral retinal dystrophy (Fig. 3C, D). Post-axial polydactyly of both the upper limb (Fig. 3A) and the left lower limb (Fig. 3B) was noted. The rest of her physical examination was unremarkable.

Laboratory investigations revealed hyperglycemia, metabolic acidosis, and ketonuria. HbA1C was 14.5%. She was diagnosed with BBS because she had four major features (retinal dystrophy, post-axial polydactyly, obesity, and learning impairments), as well as three secondary features (developmental delay, diabetes mellitus, and strabismus).

For more information, see Tables 1 and 2.

On the basis of her clinical presentation, diabetic ketoacidosis was diagnosed. Intravenous fluids, short-acting insulin, and potassium replacement were given to the patient in the intensive care unit. Her parents were advised to schedule routine checkups, and she was started on insulin after consulting with an endocrinologist.

### Case 4

A 16-year old Sudanese male, outcome of normal vaginal delivery, the fifth issue of consanguineous marriage, came to a university hospital in Sudan complaining of blurring of vision, of gradual onset, especially at night, since the age of 7 years. He had delayed developmental milestones compared with his siblings. Now he is in grade 3, primary school, and his school performance is poor. He was diagnosed with cataracts at the age of 11. There was no history of diabetes, hypertension, chronic kidney disease, or other chronic conditions. Obesity, learning difficulties, polydactyly, and vision impairment in his sister, as well as diabetes mellitus in his mother, are significant aspects of his family history.

**Fig. 4:**
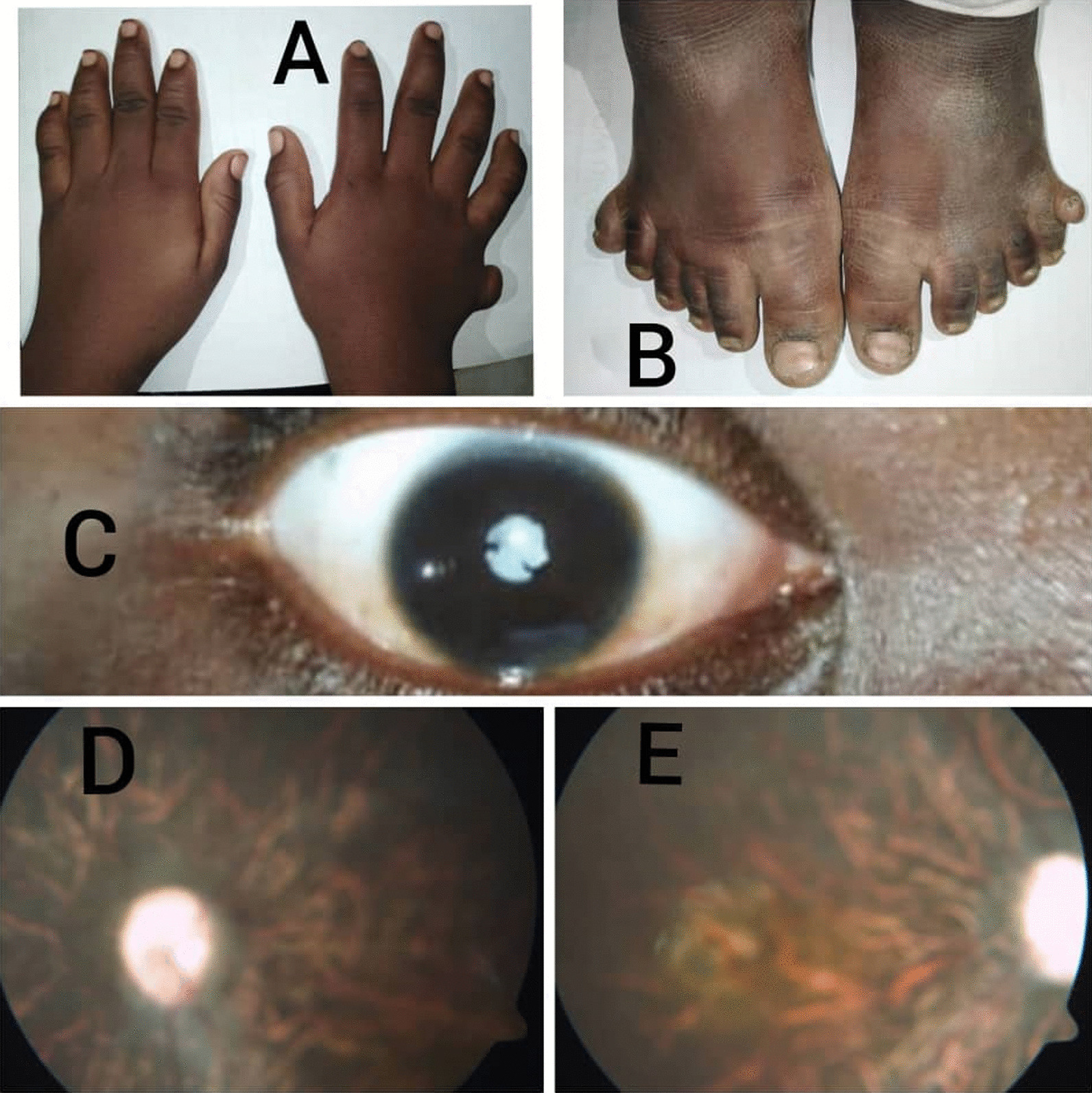
**A** Right upper limb post-axial polydactyly. **B** Bilateral lower limbs post-axial polydactyly. **C** Slit lamb examination of the right eye showing opacity of the right lens (cataract). **D**, **E** Funduscopic pictures of both eyes showing retinal dystrophy

On physical examination, the patient’s weight was 44 kg and his height was 122 cm, with a BMI of 31.1 kg/m^2^, indicating obesity. The vital signs were normal. He scored 70 on the Wechsler adult intelligence scale. Ophthalmic examination showed opacity of the right lens (Fig. 4C) with a decreased visual acuity of 20/120 in the right eye. Fundus examination showed bilateral retinal dystrophy (Fig. 4D, E). Post-axial polydactyly of both the lower limbs (Fig. 4A) and the right upper limb (Fig. 4B) was noted. Hepatomegaly was discovered during an abdominal examination.

The diagnosis of BBS was reached as the patient showed four major features (retinal dystrophy, post-axial polydactyly, obesity, and learning disabilities) plus two secondary features (developmental delay and cataract).

Table 2 contains the findings of the patient’s laboratory tests and imaging studies.

## Discussion

Bardet–Biedl syndrome (BBS) is a rare genetic multisystem ciliopathy. It has an autosomal recessive pattern of inheritance. The estimated incidence of BBS is 1 in 150,000–160,000 in North American and European populations, but the incidence appears to be higher in areas with prominent levels of consanguine marriage [5].

Over the past 10–15 years, cilia loss and/or dysfunction have been linked to numerous human disorders, collectively termed ciliopathies. BBS falls under the spectrum of ciliopathy disorders, where the function of various ciliated systemic organs is disturbed, resulting in systemic manifestations.

BBSomes are proteins that are encoded by Bardet–Biedl genes. These BBSomes function to promote the biogenesis and functions of the primary cilia.

Twenty-one BBS genes (BBS1–BBS21) have been identified. The two main genes involved in BBS are BBS1 and BBS10, which are present in more than 20% of the cases. Patients with mutations in BBS1 generally present later than patients with mutations in BBS10, owing to a milder phenotype and a later onset of retinal degeneration. As long as genetic analysis is expensive and time-consuming, it is restricted to difficult cases and research purposes. BBS can be diagnosed by clinical criteria alone.

The primary clinical features of BBS include rod–cone dystrophy, post-axial polydactyly, central obesity, cognitive impairment, hypogonadism, complex genital anomalies, and renal dysfunction [8]. The secondary features include speech disorders or delays, eye abnormalities like strabismus, cataract, and astigmatism, brachydactyly or syndactyly, developmental delay, ataxia, diabetes mellitus, craniofacial dysmorphism, nephrogenic diabetes insipidus, hepatic fibrosis, and left ventricular hypertrophy/congenital heart disease [4, 9].

The presence of four primary features or three primary and two secondary features is required to diagnose BBS. According to a large population-based survey done in the UK, the average age at diagnosis was 9 years.

In the literature, a wide range of renal abnormalities have been reported in BBS, including CKD, parenchymal cysts, calyceal clubbing, fetal lobulation, renal scarring, unilateral agenesis, dysplastic kidneys, renal calculi, and vesicoureteric reflux [10]. The natural history of renal disease in BBS is still questionable. Renal impairment can be caused by either primary causes (for example, cystic renal disease) or secondary causes (hypertension, diabetes, or metabolic syndrome). Renal failure is the most common cause of death in patients with BBS, accounting for 25% of deaths by the age of 44 [11]. The management of renal failure due to BBS is similar to other situations. All three modalities of renal replacement therapy, that is, chronic peritoneal dialysis, hemodialysis, and renal transplantation, can be applied to patents with BBS [12, 13].

In this report, the first and second cases presented with chronic kidney disease.

Studies showed an increased prevalence of insulin resistance and metabolic syndrome in adult patients with BBS compared with the general population, which is in keeping with increased cardiovascular mortality. However, overt diabetes has not yet developed in many subjects, and this may represent an opportunity to intervene with lifestyle measures [14]. Loss of BBS genes, *BBS1* or *BBS4*, resulted in a significant increase in pancreatic β-cell production.

Evaluation strategies to identify patients with BBS involve medical history, family history, physical examination, laboratory testing, and genetic testing. Regarding the medical history, the diagnosis of BBS should be considered in any individual with any of the major features listed in Table 1. The presence of these features should prompt a broad evaluation, which may disclose a BBS diagnosis.

Further, BBS should be considered in a fetus or infant with structural kidney abnormalities, genitourinary malformations, and/or polydactyly, as these findings may be the only evidence of BBS in this age group. Central obesity, a noticeable early feature of BBS, usually develops in the first year of life. Features of cone–rod dystrophy (photophobia, reduced visual acuity, and loss of color discrimination) and chronic kidney disease (polyuria and polydipsia) are usually detected by school age, whereas features of hypogonadism (lack of pubertal development) may not be detected until early adolescence. To date, there is no curative treatment for BBS. Early diagnosis is necessary to guide the patient’s follow-up through regular assessment [3, 4].

BBS requires a multidisciplinary team of pediatricians, nephrologists, orthopedic surgeons, cardiologists, ophthalmologists, dental specialists, speech pathologists, and audiologists to manage. Considering cone–rod dystrophy, early educational plans should be adopted on the basis of the certainty of blindness. Regarding education, the use of Braille, mobility training, adaptive living skills, and computing skills (including voice recognition and transcription software), in addition to the use of large-print reading materials while vision is still present, is important [12, 13].

Concerning obesity, a healthy, carbohydrate-reduced diet and regular aerobic exercise, such as walking and cycling with adaptations for blindness, are advised to control obesity. The presence of a companion to support fitness and a healthy diet can be helpful. As in our patients, the lack of well-trained dietitians in resource-limited areas and blindness poses a big challenge in diet control for patients with BBS. Metabolic syndrome and the other obesity-related complications of BBS should be managed as in the general population. For cosmetic purposes, the surgical removal of accessory digits can be done [15]. Anosmic patients with an absent or reduced sense of smell should have alternative ways of identifying dangerous materials, such as spoiled food and smoke. Learning disabilities may necessitate entrance to special schools and educational programs. Finally, genetic counseling can be of great benefit to affected individuals and their families [12, 13].

### Limitations

A genetic study to validate the diagnosis of BBS was not conducted because of its unavailability in Sudan.

## Conclusion

BBS is a rare clinical syndrome that may go unnoticed by many clinicians. Renal failure is the leading cause of morbidity and mortality in patients with BBS. Therefore, early detection of BBS is vital to halt the progression of renal impairment. The combined presence of post-axial polydactyly, blindness, learning disabilities, renal malformations, and obesity in a patient should alert to the possibility of BBS.

## Data Availability

The data used in this report are available to readers.

## Abbreviations

BBS: Bardet–Biedl syndrome
BMI: Body mass index
BP: Blood pressure
CKD: Chronic kidney disease
LVH: Left ventricular hypertrophy
